# Spiking without Resets: Continuous Integrate-and-Fire Dynamics in Neuronal Circuits

**DOI:** 10.1021/acs.jpclett.6c01632

**Published:** 2026-06-29

**Authors:** Roberto Fenollosa, Juan Bisquert

**Affiliations:** Instituto de Tecnología Química (ITQ), Consejo Superior de Investigaciones Científicas-Universitat Politècnica de València, 46022, València, Spain

## Abstract

The leaky integrate-and-fire paradigm is widely used to describe spiking dynamics in biological and artificial neurons. However, its implementation typically relies on explicit reset rules or intrinsic mechanisms involving negative differential resistance. Here we address the question: Can spike-like behavior emerge within a fully continuous dynamical framework without such ingredients?. We study a minimal resistive–capacitive circuit coupled to a conductance-activated quasi-linear memristor characterized by a single intrinsic relaxation time scale and an internal state variable. We demonstrate that spiking arises from the nonlinear coupling of the state variable and its voltage-dependent equilibrium value. Rather than being governed by a minimum transition slope in the static current–voltage characteristics, the onset of spiking does not exhibit sharp parametric thresholds but instead depends sensitively on the excitation frequency.

## Graphical abstract

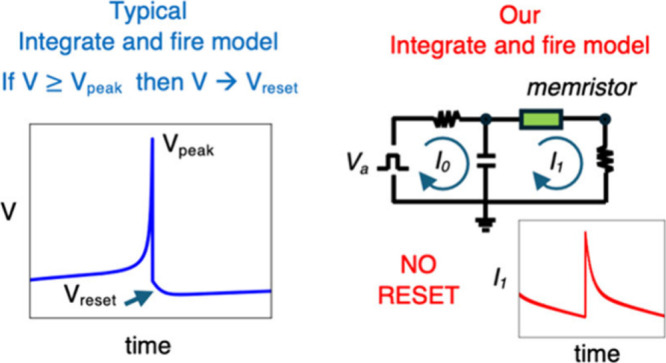

The leaky integrate-and-fire (LIF) model has played a central role in neuronal modeling since its introduction by Lapicque in 1907.^1,2^ In its classical formulation, the neuronal membrane is represented as a parallel resistor–capacitor circuit, where the membrane potential integrates an incoming DC current until a threshold is reached, at which point an instantaneous spike is emitted and the voltage is reset. Importantly, the subthreshold dynamics are described by a single linear differential equation, whereas the spike itself is not generated by the governing dynamics. Instead, both spike emission and reset are imposed externally through ad hoc rules.

Subsequent refinements^3–5^ improved the ability of LIF models to reproduce neuronal firing patterns. In particular, quadratic LIF models^6^ introduced nonlinear dynamics capable of generating diverging trajectories that resemble action potentials. Nevertheless, an explicit reset mechanism remained indispensable. Unlike the original LIF model, where the reset is required to sustain repetitive firing, in these nonlinear variants the reset primarily serves to limit spike amplitude and restore the system to a physiologically relevant state. Despite this improvement, spike termination and reinjection into the firing cycle remain externally imposed operations rather than consequences of the continuous dynamics.

This conceptual separation between subthreshold integration and spike generation has profoundly influenced theoretical neuroscience. However, it also imposes a structural limitation: spiking is not an emergent property of the continuous dynamics but an externally imposed event. In contrast, biophysically detailed models such as Morris–Lecar^7^ or Hodgkin–Huxley^8,9^ generate spikes self-consistently, yet at the cost of increased dimensionality and complexity. In fact, these models may be viewed as comprising two distinct dynamical components: a subthreshold regime that closely resembles the classical LIF description and an additional mechanism responsible for spike generation.^10^ From a dynamical systems perspective, sustained oscillations are typically associated with at least two state variables and the presence of negative differential resistance (NDR)^11–15^ or an equivalent feedback mechanism capable of supporting limit cycles.^16–20^

Recently, the LIF paradigm has been extended to threshold memristive circuits for neuromorphic and unconventional computing applications.^21–23^ These systems typically lack intrinsic NDR, yet spiking behavior has been experimentally demonstrated under pulsed excitation. However, in such studies either a detailed dynamical description was absent or the proposed models still relied on explicit reset operations or piecewise-defined dynamics enforcing a discontinuous return to a prescribed state.^5,24,25^ Consequently, whether repetitive spiking can emerge from a fully continuous low-dimensional dynamical system without resets or intrinsic NDR has remained an open question.

Here, we show that this widely accepted dichotomy is not a necessary property on fundamental grounds. We demonstrate that regular spiking can emerge in a fully continuous dynamical system of minimal dimensionality, without invoking piecewise resets or intrinsic NDR, provided that the excitation is pulsed. The mechanism relies on the interplay between the memristive internal state and the charging dynamics of a simple RC circuit, giving rise to self-consistent spike generation. The resulting dynamics reproduce the essential features of integrate-and-fire behavior while preserving continuity and involving only a single internal state variable governed by one intrinsic memory time scale. These results demonstrate that reset operations are not a fundamental requirement for integrate-and-fire behavior but rather a modeling convenience adopted in traditional formulations.

We define stable (regular) spiking as a dynamical regime characterized by well-resolved spikes emerging above the noise background, a nearly constant spike amplitude, and spiking frequency (equivalently, constant interspike intervals). Regimes that do not simultaneously satisfy these criteria are classified as irregular or suppressed spiking. Importantly, this regime is not generic in the parameter space. It arises only within a constrained region defined by coupled time scale relations among excitation frequency, duty cycle, capacitance, conductance contrast, and the intrinsic relaxation dynamics of the memristive element. Because these parameters act in a strongly interdependent manner, small variations can either suppress spiking entirely or produce irregular oscillations, thereby masking the presence of a stable LIF regime. Within this framework, we reassess the role of the memristive conductance slope during the transition from low to high conductance.^26,27^ Rather than constituting an absolute threshold parameter, its impact is inherently dynamical and is determined by its relation to the excitation frequency and the associated time scale hierarchy. This parameter sensitivity likely explains why such behavior has remained unnoticed, despite the long-standing investigation of LIF systems.

Regardless of the stringent dynamical requirements for observing LIF behavior, our findings expand the range of materials potentially suitable for neuromorphic implementations. Materials or device architectures that might have been previously disregarded—owing to insufficient conductance contrast, or modest transition slope—may nevertheless sustain robust spiking when operated within the appropriate dynamical regime. Representative examples can be found in the field of perovskite memristors,^28^ organic electrochemical transistors,^29^ and other systems.^30^

We begin by deriving an analytical formulation of the memristor model and associated system equations. On this basis, we analyze the oscillatory properties of the model and identify the dynamical constraints that enable the onset of regular spiking, determining the critical time scale relations that govern the stability and persistence of the oscillatory state.

The minimal circuit considered in this study is shown in Figure 1(a). It is composed of two resistors, a capacitor, and a memristive element (green box) that switches between high and low resistive states within a single intrinsic transition time scale.

**Figure 1:**
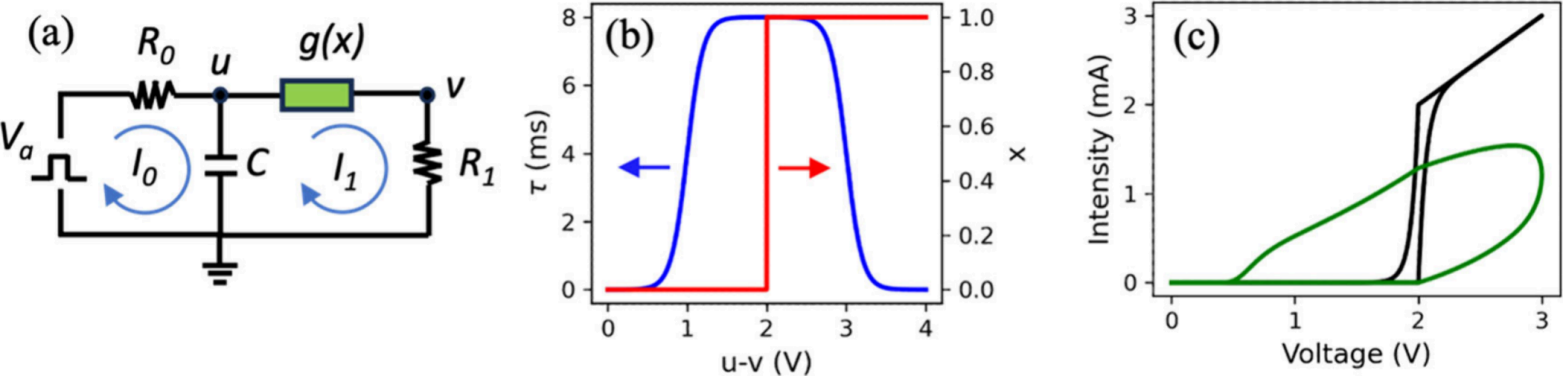
(a) Schematic of the circuit for simulating the LIF behavior. The green box corresponds to a memristor defined by an internal variable *x*. (b) Relaxation time, τ (blue curve), and memory variable, *x* (red curve), variation across the voltage difference between the two poles of the memristor according to eqs 5 and 4, respectively. (c) *I–V* characteristics obtained when scanning the memristor standalone at two different speeds: 6 V/s (black curve) and 500 V/s (green curve). Simulation parameters are *V*_1/2_ = 2 V, *V*_s_ = 10^–4^ V, τ_m_ = 8 ms, τ_0_ = 8 ms, *V*_m_ = 0.1 V, *V*_A_ = 3 V, *V*_D_ = 1 V.

The input signal *V*_a_ consists of a series of voltage pulses, and it is applied through a series resistance *R*_0_ to node *u*, which is shunted to ground by a capacitor *C*. Initially, the memristor is at its low conductance state (*g*(*x*) = *g*_L_), and therefore, the capacitor integrates the input current while simultaneously leaking through the nonlinear branch formed by the memristor in series with a load resistor *R*_1_. Once the voltage at node *u* reaches a certain value, the conductivity of the memristor increases abruptly, producing a sharp shoot of the intensity, *I*_1_, and discharging the capacitor.

To account for this behavior, we introduce a simple approach of a memristor governed by the Conductance-Activated Quasi-Linear Memristor (CALM) model.^31^ It is formulated in terms of two differential equations:

1 $$C\frac{du}{dt}=\frac{\left(V_{a}-u\right)}{R_{o}}-{\left(\frac{1}{g}+R_{1}\right)}^{-1}u$$

2 $$\tau \frac{dx}{dt}=\left(x_{eq}-x\right)$$

The first differential equation of the system is derived by applying Kirchhoff’s current law at node *u* [eq 1]. The second differential equation accounts for the fact that the internal state variable of the memristor, *x*, does not evolve instantaneously but instead changes according to a characteristic relaxation time, τ [eq 2]. The effective memristor conductance is defined as

3 $$g=g_{L}+\left(g_{H}-g_{L}\right)x$$

with *g*_L_ and *g*_H_ representing the low- and high-conductance states, respectively.

The equilibrium value of the state variable is described by a sigmoidal function of the memristor terminals voltage (*u – v*):

4 $$x_{eq}=\frac{1}{1+e^{-\left(u-v-V_{1/2}\right)/V_{S}}}$$

where *V*_1/2_ is the midpoint switching voltage and *V*_S_ controls the steepness of the transition. Equation 3 provides a linear character to the system, while eq 4 introduces a nonlinear component.

The time constant τ is voltage-dependent according to the following equation:

5 $$\frac{1}{\tau }=\frac{1}{\tau_{m}}+\frac{1}{\tau_{0}}\left(e^{\left(u-v-V_{A}\right)/V_{m}}+e^{-\left(u-v-V_{D}\right)/V_{m}}\right)$$

This equation exhibits a Gaussian-like profile [Figure 1(b)] and is motivated by the exponential voltage–time relationship commonly observed in memristive switching processes governed by ionic transport and filament formation.^30,32^ The parameters *V*_D_ and *V*_A_ define a voltage window where the relaxation time is enhanced and memory effects become significant, whereas outside this interval the relaxation time decreases rapidly and the device responds almost instantaneously. The parameter τ_m_ sets the maximum relaxation time, τ_0_ controls the relaxation-time decay outside the memory window, and *V*_m_ determines the sharpness of the transition between volatile and memory-retaining regimes. As a consequence, the standalone *I*–*V* characteristics of the memristor depend strongly on the voltage scanning speed [Figure 1(c)]. This dependence is particularly relevant because the effective slope experienced by the system during the initial stages of the transition from the low- to the high-conductance state is governed by the rate at which this transition point is approached.

We now show the oscillatory properties of the NDR-free model. The LIF regime is characterized by a sharp increase in the current flowing through the circuit branch containing the memristor, denoted as *I*_1_. This behavior is illustrated in Figure 2(a), where, in addition to *I*_1_, we have plotted the temporal evolution of other variables, specifically *u – v*, *x*_eq_, and *x*. Figure 2(b) presents a zoomed view of a single spike; in this panel, the square excitation pulse *V*_a_ is also plotted together with *u – v*. The parameters utilized for this simulation are detailed in the figure’s caption.

**Figure 2:**
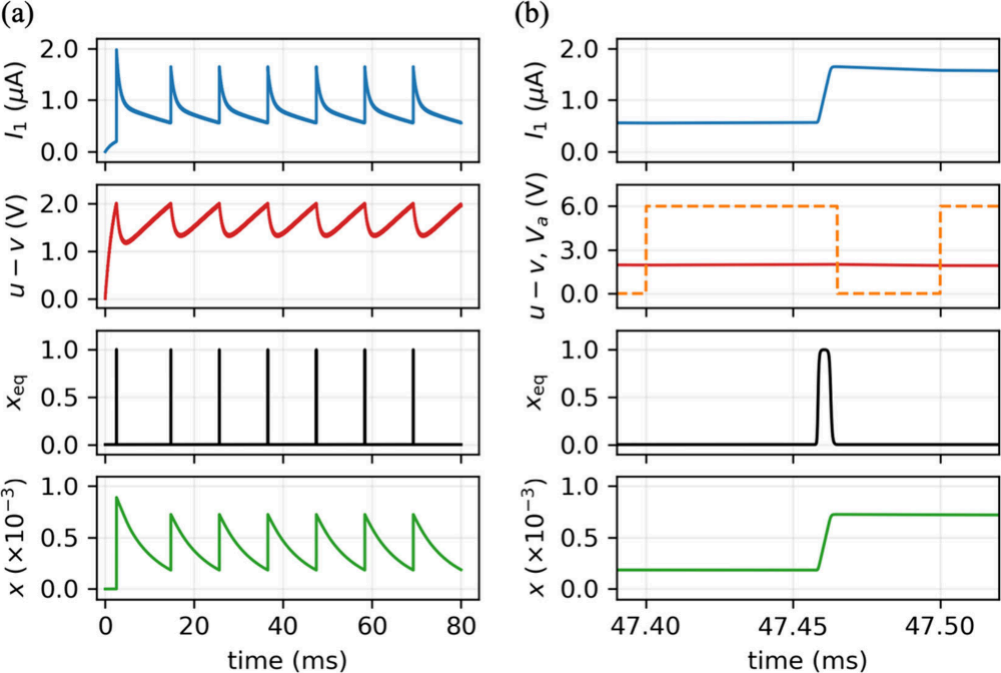
(a) Time evolution of several variables of the system of Figure 1(a) illustrating the LIF behavior. (b) Same as (a) but in a zoomed area. In addition, the excitatory signal *V*_a_ is represented together with *u – v* to illustrate the pulse excitation duration. The simulation parameters are *V*_1/2_ = 2 V, *V*_s_ = 10^–4^ V, τ_m_ = 8 ms, τ_0_ = 8 ms, *V*_m_ = 0.1 V, *V*_A_ = 3 V, *V*_D_ = 1 V, *g*_L_ = 10^–7^ S, *g*_H_ = 10^–3^ S, *R*_0_ = 3 MΩ, *R*_1_ = 1 kΩ, *C* = 1 nF, *V*_a_ = 6 V, *f* = 10 kHz (excitation pulse frequency), and *D* = 0.65 (duty cycle of the pulse).

Figure 2 shows that a standard threshold memristor without an NDR is able to be driven to oscillations by a pulsed external signal. The constrained nature of this regime becomes evident when the parameter space is explored in detail. Rather than emerging through a simple bifurcation or a single control parameter, regular spiking results from a delicate balance between coupled time scales. When the coupled time scale relations are not satisfied, the system typically fails to exhibit regular spiking and instead either remains nonoscillatory or develops irregular spike trains with fluctuating amplitudes and latencies.

What should be the system properties to obtain oscillations? The first important practical criterion was already pointed out in previous studies.^21^ It concerns the charge and discharge times occurring in both branches of the system, that are characterized by intensities *I*_0_ and *I*_1_, respectively [Figure 1(a)]. During the charging stage of the capacitor, its charging time is characterized by the value *R*_0_ *C*, and it should be shorter than the discharging or leaking time, that can be evaluated as (*R*_1_ + 1/*g*_L_)*C*; otherwise the capacitor could not be charged. However, during the spike onset, the discharging time, (*R*_1_ + 1/*g*_H_)*C*, must be much shorter than the charging time. In other words, the charging time of the capacitor should have a value within the two extreme time values given by the low and high conductance states of the memristor. In the simulation of Figure 2, these times are 3, 10 and 0.002 ms, respectively. In addition, the time, τ_m_, that determines the bell maximum of τ [blue curve in Figure 1(b)] must have at most a value within the same order of magnitude as that of the capacitor charging time, *R*_0_ *C*. Otherwise, the voltage dynamics would evolve much faster than the internal memory state, weakening the coupling between both variables and suppressing the integrate-and-fire mechanism. All in all, the period of the spikes is going to be influenced by all these times. Particularly, τ_m_ can be used for tuning this period as shown in Figure 3. Nevertheless, it should be mentioned that the spiking period is related to but not equal to τ_m_.

**Figure 3:**
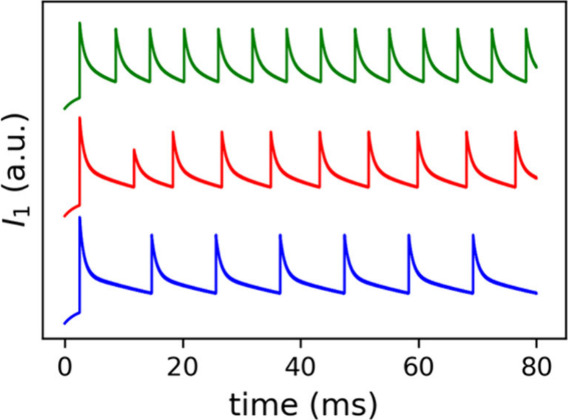
Time evolution of *I*_1_ for different values of τ_m_: 8 ms (blue), 6 ms (red) and 4 ms (green). They produce spikes with periods of 10.9, 8.3, and 5.8 ms, respectively. The other simulation parameters are similar to those of Figure 2.

In essence, the emergence of an LIF process requires a pronounced contrast between fast and slow variations in the memristor conductance *g*. As mentioned above, in many previous approaches, this abrupt transition has been introduced artificially by incorporating discontinuities in the governing equations, forcing a state variable to reset once a prescribed condition is met. In contrast, the dynamical equation governing the internal state variable of the memristor contains only a single intrinsic time scale, τ_m_, associated with the dynamics at the transition point. At first sight, this appears incompatible with the coexistence of distinct fast and slow events required for LIF behavior.

We find that an effective fast–slow contrast emerges naturally from the mismatch between the instantaneous target value *x*_eq_ and the actual state variable. Sharp spikes in *x*_eq_, occurring only within a restricted parameter region, drive a rapid increase of *x* over the intrinsic time scale τ_m_. Because these spikes are short-lived, *x* remains far below the peak of *x*_eq_ (Figure 2). After the spike, *x* relaxes toward its baseline with the same intrinsic time scale but with a much smaller driving difference, resulting in a slow effective decay.

Thus, despite the presence of an intrinsic relaxation time scale, the nonlinear coupling between *x* and *x*_eq_ generates an emergent fast-slow dynamical hierarchy. In this sense, the LIF regime exploits the full dynamical range of *x*_eq_ while utilizing only a small fraction of its amplitude.

The mechanism described above crucially relies on the formation of sharp spikes in *x*_eq_. It is therefore essential to determine under which dynamical conditions such spikes can arise. In this context, a second practical criterion emerges naturally from our analysis. We find that the appearance of well-defined regular spikes is linked to the rate at which the voltage difference, *u – v*, traverses the transition region. Specifically, the relevant quantity is the speed of the voltage variation, *S*_tr_ = Δ­(*u – v*)/Δ*t*, just at the transition point *V*_1/2_. This speed can be estimated as

6 $$S_{tr}∼\frac{\left(V_{a}-V_{1/2}\right)}{R_{o}C}-\frac{\left(V_{a}-V_{1/2}\right)}{\left(R_{1}+\frac{1}{g_{L}}\right)C}$$

Equation 6 captures the effective rate resulting from the simultaneous integration and leaky processes in the two circuit branches. Although this rate varies during the transition, it provides a representative estimate of the speed at which the system crosses the low- to high-conductance region. The transition width in *x*_eq_ is controlled by the sigmoid parameter *V*_s_ in eq 4. For practical purposes, we define this width as 10*V*_s_, which encompasses more than 99% of the transition. The corresponding transition time is then estimated as the ratio between this voltage interval and *S*_tr_:

7 $$t_{s}=\frac{10V_{s}}{S_{tr}}=\frac{10V_{s}}{\left(V_{a}-V_{1/2}\right)}{\left[\frac{1}{R_{o}C}-\frac{1}{\left(R_{1}+\frac{1}{g_{L}}\right)C}\right]}^{-1}$$

The condition to obtain well-defined spikes is that this time, *t*_s_, is much smaller than the excitation pulse width in at least 1 order of magnitude. Specifically,

8 $$t_{s}\ll \frac{D}{f}$$

where *f* is the pulse frequency and *D* its duty cycle. This gives a high limit for the frequency, *f*_max_:

$$f\ll f_{\max}=\frac{D\left(V_{a}-V_{1/2}\right)}{10V_{s}C}\left[\frac{1}{R_{o}}-\frac{1}{R_{1}+\frac{1}{g_{L}}}\right]$$(9)

We now illustrate how the second criterion operates in practice by examining parameters that modify the effective transition time, *t*_s_. A key quantity is the intrinsic speed *S*_tr_ [eq 6], which can be tuned through the capacitance *C*. For the parameters of Figure 2 (*C* = 1 nF), we obtain *S*_tr_ = 933 V/s, yielding a transition time *t*_s_, well below the duty-cycle duration *D*/*f*, thereby satisfying condition ([Disp-formula eq8]). Increasing the capacitance to 100 nF reduces *S*_tr_ to 9.3 V/s, causing *t*_s_ to exceed *D*/*f* (see Table 1) and consequently lowers the maximum operating frequency *f*_max_. Under these conditions, regular oscillations disappear at 10^4^ Hz [blue curve in Figure 4(a)]. This behavior directly reflects the violation of condition ([Disp-formula eq8]): as shown in Figure 4(b), the *x*_eq_ peaks are no longer well-confined within the excitation pulse, preventing the state variable *x* from experiencing a sufficiently localized driving stimulus. As a consequence, the system does not follow a reproducible dynamical trajectory from one cycle to the next, leading to fluctuations in the spike amplitude and timing.

**Figure 4:**
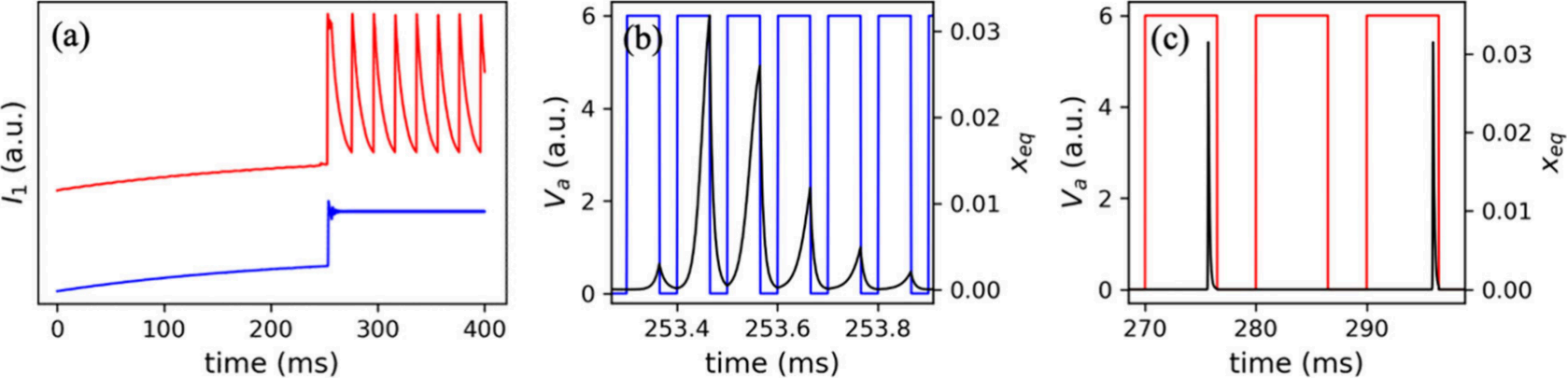
(a) Time evolution of *I*_1_ for *C* = 10^–7^ F and two different excitation frequencies: *f* = 10^4^ Hz (blue curve), *f* = 10^3^ Hz (red curve). All other parameters are identical to those of Figure 2. (b) and (c) Corresponding excitatory pulsed signals (blue and red curves, respectively) and *x*_eq_ evolution (black curves).

**Table 1:** Values of *C* and *f* used in the Simulations of the *I*_1_ Curves of Figure 4 (All Other Parameters Are Identical to Those in Figure 2), Together with the Corresponding Calculated Quantities Relevant to the Emergence of a Regular Spiking Regime

*I*_1_ curve	*C* (F)	*f* (Hz)	*f*_max_ (Hz)	*f*_min_ (Hz)	*S*_tr_ (V/s)	*t*_s_ (μs)	*D*/*f* (μs)
**Blue**	10^–7^	10^4^	6 × 10^3^	55	9.3	100	65
**Red**	10^–7^	10^2^	6 × 10^3^	0.5	9.3	100	6500

Restoring the inequality *t*_s_ ≪ *D*/*f* by reducing the excitation frequency to 10^2^ Hz [red curves in Figure 4(a) and (c)] reestablishes the oscillatory regime, even though the peak amplitude *x*_eq_ remains below its maximal value.

Another important factor influencing the time relations described above is the shape of the sigmoid curve. In particular, increasing *V*_s_ broadens the transition region and consequently increases *t*_s_ (see Table 2), resulting in a maximum frequency limit *f*_max_ of 6 × 10^4^ Hz, which becomes comparable to the excitation frequency (10^4^ Hz). This suppresses the oscillatory spiking regime. However, reducing the excitation frequency, for example, to 10^3^ Hz, restores the oscillatory behavior. This effect is illustrated in Figure 5(a) by blue and red curves, respectively.

**Figure 5:**
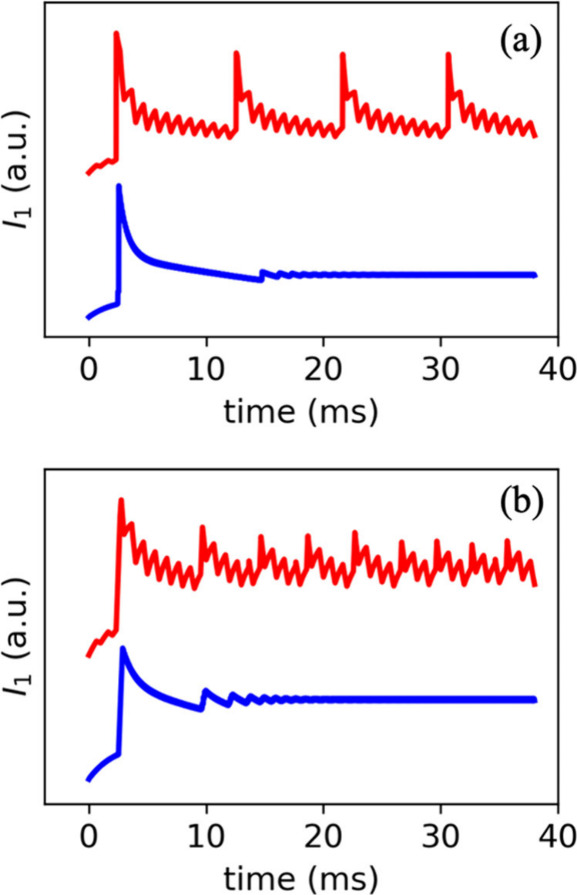
(a) Time evolution of *I*_1_ for *V*_s_ = 0.001 V and two different excitation frequencies: 10^4^ Hz (blue curve) and 10^3^ Hz (red curve). The other parameters are similar to those of Figure 2. (b) Same as (a) but for *g*_H_ = 10^–5^ S and *V*_s_ = 0.0001 V.

**Table 2:** Values of *V*_s_ and *f* used in the Simulations of the *I*_1_ Curves of Figure 5(a) (All Other Parameters Are Identical to Those in Figure 2), Together with the Corresponding Calculated Quantities Relevant to the Emergence of a Regular Spiking Regime

*I*_1_ curve	*V*_s_ (V)	*f* (Hz)	*f*_max_ (Hz)	*f*_min_ (Hz)	*S*_tr_ (V/s)	*t*_s_ (μs)	*D*/*f* (μs)
**Blue**	0.001	10^4^	6 × 10^4^	55	933	10	65
**Red**	0.001	10^3^	6 × 10^4^	55	933	10	650

Although reducing the excitation frequency promotes the emergence of a regular spiking regime, it also increases the time available for the voltage variable, *u*, to evolve during each excitation cycle. As a result, small secondary peaks develop in the cycles following the primary spike. These peaks form the ripple structure observed in the red curves of Figure 5(a) and (b). While their amplitude is negligible at high frequencies, it increases progressively as the excitation frequency decreases, eventually becoming comparable to that of the primary spikes. Consequently, a lower frequency bound *f*_min_ must be identified to prevent this from occurring. The procedure used to estimate this bound is described in the SI. The resulting values are reported in Tables 1 and 2 for the corresponding cases.

Finally, the conductance contrast *g*_H_/*g*_L_ also plays an important role in determining the spiking regime. In general, larger contrasts promote the emergence of well-defined spike dynamics. A reduction of this contrast, particularly through a decrease in *g*_H_, lowers the effective target conductance set by *x*_eq_, thereby preventing the sharp increase in *I*_1_ required for spike formation. This limitation can, to some extent, be mitigated by reducing the excitation frequency, which provides additional time during the duty cycle for *x* to reach a higher value. This compensation, however, comes again at the expense of a more pronounced ripple structure [Figure 5(b)].

In summary, we have demonstrated that spike-like integrate-and-fire dynamics can emerge in a fully continuous dynamical system driven by pulsed excitation, without invoking piecewise-defined reset rules or intrinsic negative differential resistance. Although the memristor is characterized by a single intrinsic relaxation time scale, the nonlinear coupling between the state variable and its voltage-dependent target gives rise to an effective fast–slow dynamical contrast. We identify two practical criteria governing the emergence of regular spiking: the balance between charging and discharging time scales, and the relation between the transition time from low to high conductance and the duration of the excitation duty cycle. Rather than being controlled by a sharp transition slope in the static *I*–*V* characteristics, the onset of spiking is determined by the interplay between excitation frequency, duty cycle, capacitance, and conductance contrast. These results clarify the dynamical origin of spiking in threshold-like memristive systems and provide simple design guidelines for implementing neuromorphic functionalities within fully continuous models.

## Supplementary Material



## Data Availability

The data presented here can be accessed at 10.5281/zenodo.18739798 (Zenodo) under the license CC-BY-4.0 (Creative Commons Attribution 4.0 International).

## Notes

Estimation of the low frequency limit, *f*_min_ (PDF)
