# Supplementary material for: Spiking without Resets: Continuous Integrate-and-Fire Dynamics in Neuronal Circuits
Source: J Phys Chem Lett. 2026 Jun 29;17(27):7619–24. doi: 10.1021/acs.jpclett.6c01632 (PMC13359364; doi:10.1021/acs.jpclett.6c01632)
Supplement: Supplementary file 1 [file jz6c01632_si_001.pdf]

Supporting information

Spiking without resets: Continuous integrate-and-fire dynamics in neuronal circuits

Roberto Fenollosa*, Juan Bisquert
Instituto de Tecnología Química (ITQ), Consejo Superior de Investigaciones Científicas-Universitat Politècnica de València, 46022, València, Spain.

Estimation of the low frequency limit, f_{min}.

The criterion used to determine f_{min} is based on requiring that the variation of I_1 during a subsequent excitation cycle remains significantly smaller than the maximum value of I_1 attained during a spike.

We first evaluate the maximum attainable current I_{1max}. From the circuit configuration, it follows that

$$I_1 = \frac{u}{\frac{1}{g} + R_1} \qquad \text{(A1)}$$

Assuming, as a first approximation, that du/dt = 0 and $u = V_{1/2}$ at the instant when I_1 reaches its maximum, Eqs. (1) and (A1) yield

$$I_{1max} \sim \frac{V_a - V_{1/2}}{R_0} \qquad \text{(A2)}$$

Which gives I_{1max} = 1.3 μA for the parameters of Fig. 2. This value agrees well with the numerical results.

The current variation responsible for the ripple structure, ΔI_1, can be estimated from Eqs. (1) and (A1), noting that this variation occurs over a time interval $\Delta t = D/f$:

$$\Delta I_1 \sim \frac{\Delta u}{\frac{1}{g_L}+R_1} = \frac{D}{C\left(\frac{1}{g_L}+R_1\right)f}\left(\frac{(V_a - V_{1/2})}{R_o} - \frac{V_{1/2}}{\frac{1}{g_L}+R_1}\right) \qquad \text{(A3)}$$

Therefore, the condition ensuring that the ripple amplitude remains small compared to the peak current is:

$$\Delta I_1 << I_{1max} \tag{A4}$$

Using Eqs. (A2) and (A3), the condition in Eq. (A4) can be reformulated as a minimum frequency constrain, f_{min}, which reads

$$f >> f_{min} = \frac{D\, R_0}{C\left(\frac{1}{g_L}+R_1\right)\left(V_a-V_{1/2}\right)}\left(\frac{\left(V_a-V_{1/2}\right)}{R_0}-\frac{V_{1/2}}{\frac{1}{g_L}+R_1}\right) \tag{A5}$$
